# P02-08 This has just given me life back’ – mixed method evaluation of the Wild Skills, Wild Spaces ecotherapy project, Wales, UK

**DOI:** 10.1093/eurpub/ckac095.027

**Published:** 2022-08-29

**Authors:** Diane Crone, Paul Sellars, Debbie Clayton, Jenny Mercer

**Affiliations:** Cardiff Metropolitan University, Cardiff, United Kingdom

**Keywords:** mental health, social inclusion, green space, conservation, purposeful activity

## Abstract

Wild Skills Wild Spaces (WSWS) is a Welsh Government funded project which aims to deliver and evaluate an ecotherapy programme to improve the health, skills, and wellbeing of local communities in rural Montgomeryshire, Wales. Montgomeryshire Wildlife Trust (a third sector organisation) deliver the programme which is evaluated by Cardiff Metropolitan University.

The 2-year project, which began in April 2020, involves the running of 12-week interventions of weekly physically active ecotherapy activities such as tree pruning, woodwork, growing vegetables, walking and bushcraft skills. Participants (n = 40; mean age = 24.75 *SD* = 15.22) are people with mental health and/or behavioral problems referred via the National Health Service, health and social care partners, and schools.

The mixed method evaluation includes two phases: (i) a quantitative phase which involves pre and post intervention measures concerning connectedness to nature, wellbeing, and physical activity level; and, (ii) a qualitative phase which involves interviews and focus groups with participants, referrers, and programme leaders.

At this early stage in the data collection, to date no significant changes in pre and post ecotherapy intervention quantitative scores have been apparent. However, 100% of respondents have stated that they would like to continue taking part in ecotherapy programme. From the qualitative phase, participants described positive experiences of the programme particularly referring to how the programme supported their mental health, wellbeing, and social interactions. Additionally, participants highlighted the importance of the programme leaders in facilitating an enjoyable and safe environment in which they could relax, interact with others, and learn new skills.

Findings from year 1 of the 2-year evaluation provide promising results that ecotherapy programmes have the potential to attract socially excluded individuals into purposeful programmes. These programmes appear, through the qualitative findings to date, to benefit participants' self-reported holistic health but are also valued by them, for their contribution to supporting the environment through playing a part in the management of outdoor, green space in our communities.

